# Delta waves as a sign of cortical plasticity after full-face transplantation

**DOI:** 10.1038/s41598-024-67469-w

**Published:** 2024-07-16

**Authors:** Esra Süzen, Ayhan Şavklıyıldız, Ömer Özkan, Ömer Halil Çolak, Ebru Apaydın Doğan, Özlenen Özkan, Buket Şimşek, Ümit Deniz Uluşar, Hamza Feza Carlak, Övünç Polat, Hilmi Uysal

**Affiliations:** 1 Faculty of Engineering, Department of Electrical and Electronics Engineering, Akdeniz University, Pınarbasi Blvd., Antalya, Turkey; 2 Faculty of Medicine, Department of Plastic and Reconstructive Surgery, Akdeniz University, Antalya, Turkey; 3 Faculty of Medicine, Department of Neurology, Akdeniz University, Antalya, Turkey; 4 Faculty of Engineering, Department of Computer Engineering, Akdeniz University, Antalya, Turkey

**Keywords:** EEG, Transplants, Facial lesions, Wavelet, Delta band, Brain plasticity, Biomedical engineering, Neurology, Neuroscience, Computational neuroscience

## Abstract

This study focused on detecting the reflections of healing and change in cortex activation in full-face transplantation and lesions patients on EEG activity. Face transplant patients have facial lesions before transplantation and, to identify pre-face transplant patients' brain activity in the absence of pre-transplant recordings, we used data obtained from pre-transplant facial lesion patients. Ten healthy, four facial lesion and three full-face transplant patients participated in this study. EEG data recorded for four different sensory stimuli (brush from the right face, right hand, left face, and left-hand regions) were analyzed using wavelet packet transform method. EEG waves were analyzed for standard bands. Our findings indicate significant change in the 2–4 Hz frequency range which may be a result of ongoing or previous cortical reorganization for face lesion and transplant patients. Alterations of the delta wave seen in patients with facial lesion and face transplant can also be explained by the intense central plasticity. Our findings show that the delta band differences might be used as a marker in the evaluation of post-transplant cortical plasticity in the future.

## Introduction

The process of replacing all or part of the human face with facial tissue taken from a suitable donor by surgical operation is called face transplant. Face transplant is classified as a vital transplant as it improves the quality of life^1^. This type of surgical operation has been performed in various countries since 2005^2–6^. Ballistic trauma and burn patients account for two-thirds of all transplant recipients worldwide^7^. Lesions have different formation characteristics and differ from each other in location, depth and size. In patients with full-face transplants and facial lesions, changes in the cerebral cortical reorganization, as well as functionality and structure of the face, are observed. Traumatic loss of facial functions with loss of facial structures (skin, muscles, nerves, and bones) cause reorganizations in the primary motor and somatosensory cortex^8^. Facial skin has the highest concentration of sensory receptors in the body^1,9^. Therefore, in response to the sudden and prolonged loss of the facial structures brain reorganizes^10–12^. This reorganization, defined as brain plasticity, has also been observed in clinical examinations in face transplant patients^13–15^.

As the number of patients is increasing day by day, reconstructing the facial anatomy and functions of full-face transplant patients become important^16^. In patients with full face transplantation, the functionality and sensory modalities of the face are lost over time after the damage, and after the transplantation sensory patterns begin to recover in a short time. Therefore as Siemionow mentioned in their study, it is important to examine lost modalities and the process of regaining them^17^. Therefore, defining this relationship in face transplantation is also very valuable for surgeons in the field of composite tissue allograft transplantation^18,19^. Functional mapping of the brain is important to elucidate this issue. In a study carried out by this team, it was found that muscle activities of the patients who underwent face transplants differ in frequency spectrum compared to the healthy group^20^. In addition, studies including motor activities developed with cognitive rehabilitation^21^ suggest that changes in the somatosensory cortex in pre-transplant cases may also be important to understand the progress of patients' recovery and to be able to model them in the future. Phantom sensation is the feeling of a stimulus in a region other than where it is originated^22–25^. In a study conducted with clinical examination of face transplant patients, the regional sensation was defined in different areas of the face as a result of facial and hand stimuli, and it was shown that there is a correlation between fMRI cortical representation^13–15^. The most important feature of the full-face lesion patients is that the residual tissues of the face are different from each other, and when combined with plasticity, the brain organization can reveal patient-specific differences. Determining the changes in EEG sub-frequencies caused by brain plasticity resulting from limb loss in terms of cortex activation is critical.

In this study, which was performed on full-face lesion and transplant patients, cortex reorganization was examined using EEG, and the identification of these reorganizations were investigated with EEG waves as well as physician observations. Study by Siemionow et. al. is the only available study in EEG literature, which compared a case with facial lesions (pre-transplant) and a full-face transplant (post-transplant) patient^26^.

In the analysis of EEG signals, standard practice involves examining the main frequency bands (delta, theta, alpha, beta, gamma). Instead of using the main bands of the EEG, dividing them into sub-bands might be valuable for producing more effective results. This approach is also applied in various studies in the literature. For example, Dumas and his colleagues showed new evidence for autism spectrum disorder by segregating the alpha-mu rhythm into two sub-bands^27^. In another study in the field of anesthesia, a sub-band of the delta, the fast delta [2–4 Hz], might be used to measure the depth of anesthesia^28^. According to a sleep EEG study, apart from dividing standard bands into two equal parts, splitting the beta band into 15–18 Hz and 18–30 Hz revealed a sharp distinction around 18 Hz and showed that different temporal patterns are exhibited in different sleep cycles^29^. Thus, dividing each main band into two sub-bands enhances frequency resolution and potentially reveals subtle changes in brain activity that might be missed with broader band analysis.

Different than the available studies, in this study, changes in EEG sub band activity were investigated and discussed according to both patient type with a large patient group including cases with fullface lesions and full-face transplants. In the cortical map, the hand and face regions are adjacent to each other^30–35^. Therefore, this study focused on two different stimulus regions, hand and face. As a result of the analyzes, distinctive features were determined for both patient groups and for different stimulus types, and the results were evaluated and interpreted in the discussion section.

## Materials and methods

### Participants

Four facial lesion and three full-face transplant patients participated in this study. Ten healthy volunteers without a history of neurological or psychiatric disorders were selected as the control group. Consents of subjects were obtained from all participants. This study was approved by Akdeniz University, Faculty of Medicine, Clinical Research Ethics Committee (2012-KAEK-20/996, 23.10.2019). Patient information is given in Table 1.

**Table 1:** Participant information.

Code	Age	Sex	Patient Story	Surgery Operation	EEG recorded time after the operation
Cx	24.2 ± 5.2	7 M–3 F	Healthy	No operation	
T1	24	M	Burn (1yo)	Full face transplant (19yo)	5 years later
T2	40	M	Burn in infancy (3yo)	Full face transplant (35yo)	5 years later
T3	30	M	Gun accident (6yo)	Full face transplant (26yo)	4 years later
L1	18	M	Burn in infancy (10mo)	Correction of scars (18yo)	6 months later after the last operation
L2	46	M	Gun accident (45yo)	V–Y advancement skin flap from the right oral commissure (45yo)	1 year later
L3	22	F	Burn in infancy (2mo)	Multiple Skin grafting and scar revision sessions (over the years)	6 years later after the last operation
L4	14	F	Burn in infancy (9mo)	Multiple Skin grafting and scar revision sessions (over the years)	1 year later after the last operation

*T* full-face transplant, *L* facial lesion patient, *Cx* control group, *F* female, *M* male.

Details of patient group participants have been reported in our previous studies^14,20,36^. All face transplant recordings were taken after the surgeries. Since there were no recordings after the transplants facial lesion group can be seen as pre-state of the transplants.

### Signal acquisition and experimental procedure

All recordings were taken with Nihon Kohden EEG device in the EEG laboratory in Akdeniz University Faculty of Medicine, Department of Neurology. The recordings were taken as unipolar at a sampling frequency of 200 Hz using 64-channel EEG cap consisting of Ag/AgCl electrodes specifically designed in accordance with the international 10–10 system. The cap was placed on the scalp of the individual with reference to the nasion, inion and preauricular points. Electrode gel was used to lower the skin impedance and increase conductivity. The technical specifications of the recording device are as follows: electrode impedance 100–5000 Ω, high-frequency (high pass) filter, 70 Hz, low-frequency (low pass) filter, 1 Hz, notch filter, 50 Hz and initial sensitivity in acquisitions is 7 μV/mm.

**Figure 1:**
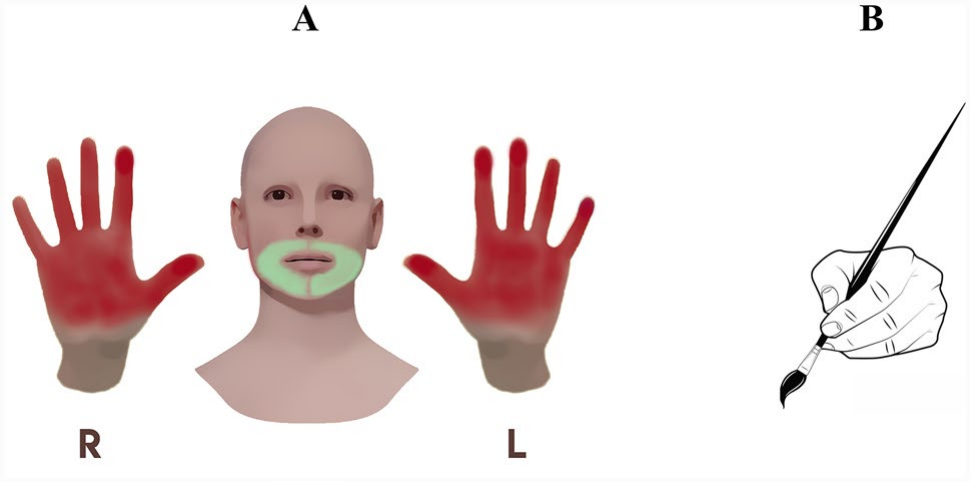
(**a**) Stimulus areas and (**b**) brush. Stimulus area illustrations were generated in Adobe Illustrator CS6 (https://www.adobe.com/tr/products/illustrator.html) combined hand with paint brush image which is public (URL link). Then figures re-edited in GIMP software 2.10.36 version (https://download.gimp.org/mirror/pub/gimp/v2.10/windows/).

In order to detect EEG signals in the resting state of the individuals participating in the study, EEG signals recorded with eyes closed for 2 min, eyes open for 1 min, and finally eyes closed for 2 min. The regions of hand and face sensory stimulations were denoted in Fig. 1. The hand stimulus was performed on the palmar surface of the right and left hands, starting from the thumb to the little finger. Facial stimuli were applied with the help of a brush on the right and left lower face regions, except for the lip region. The EEG recording procedure for hand and face stimulations were recorded with a block design. The design contained one minute of stimulation and 30 s of rest. Subjects were asked to close their eyes and stay in a comfortable and stable position during the recording.

### EEG signal processing and analysis

Wavelet packet transform was used to analyze the EEG signal. Wavelet packet transform is a generalization of discrete wavelet transform. The wavelet packet function is defined as follows.

1 $$W_{m,j,n} (t) = 2^{{ - {\raise0.5ex\hbox{$\scriptstyle m$} \kern-0.1em/\kern-0.15em \lower0.25ex\hbox{$\scriptstyle 2$}}}} W_{j} (2^{ - m} t - n)$$

where $j\in N$, $(m,n) \in Z^{2}$ and $j$ is the node index at each level, n is the time axis shift parameter, and m is the scaling parameter.

The energy in the wavelet packet calculated with the root mean square (RMS) values from the reconstruction of W_m,j,n_^37^. The RMS value for each node is calculated using Eq. (2).

2 $$w_{RMS,m,j} = \sqrt {\frac{1}{N}\sum\limits_{n = 0}^{N - 1} {\left| {w_{m,j} (n)} \right|}^{2} }$$

where j ∈ N denotes the node index in each m level.

A 50 Hz notch filter is used to eliminate the noise originating from the mains and other sources. Using wavelet packet transform (WPT), the signals are decomposed into 9 levels of 512 nodes. In order to minimize the differences and losses in the EEG band boundary frequencies, 9-level decomposition is used. As a result of decomposition with WPT, the frequency ranges in the EEG sub-bands correspond to the following nodes. Delta band 1–10 nodes 0–4 Hz (0–3.90 Hz) frequency range, Theta band 11–18 nodes 4–7 Hz (3.90–7.03 Hz), Alpha band 19–33 nodes 7–13 Hz (7.03–12.89 Hz), Beta band 13–30 Hz (12.89–30.07 Hz) for nodes between 34 and 77, and Gamma band corresponds to a frequency range of 30–50 Hz (30.07–50 Hz) between 78 and 128 nodes. Next, these bands are divided into two components with the definition of lower and upper bands^38^. The intervals of the lower and upper bands are shown in Table 2.

**Table 2:** Frequency ranges of EEG bands.

EEG band	Frequency (Hz)	Nodes	Lower band frequency (Hz)	Upper band frequency (Hz)
Delta	0–4	W_9,1_–W_9,10_	0–2	2–4
Theta	4–7	W_9,11_–W_9,18_	4–5.5	5.5–7
Alfa	7–13	W_9,19_–W_9,33_	7–10	10–13
Beta	13–30	W_9,34_–W_9,77_	13–21.5	21.5–30
Gamma	30–50	W_9,78_–W_9,128_	30–40	40–50

Alpha oscillations are observed in EEG recordings taken when eyes are closed. However, various peaks similar to alpha oscillations can be seen in the frequency spectrum^36^. Peak patterns that resemble alpha oscillations on other channels and frequencies are called near harmonics. In order to reduce the near-harmonic effect defined by Albada^39^, Daubechies type with the highest filter coefficient (db32) was chosen as mother wavelet^40^.

**Figure 2:**
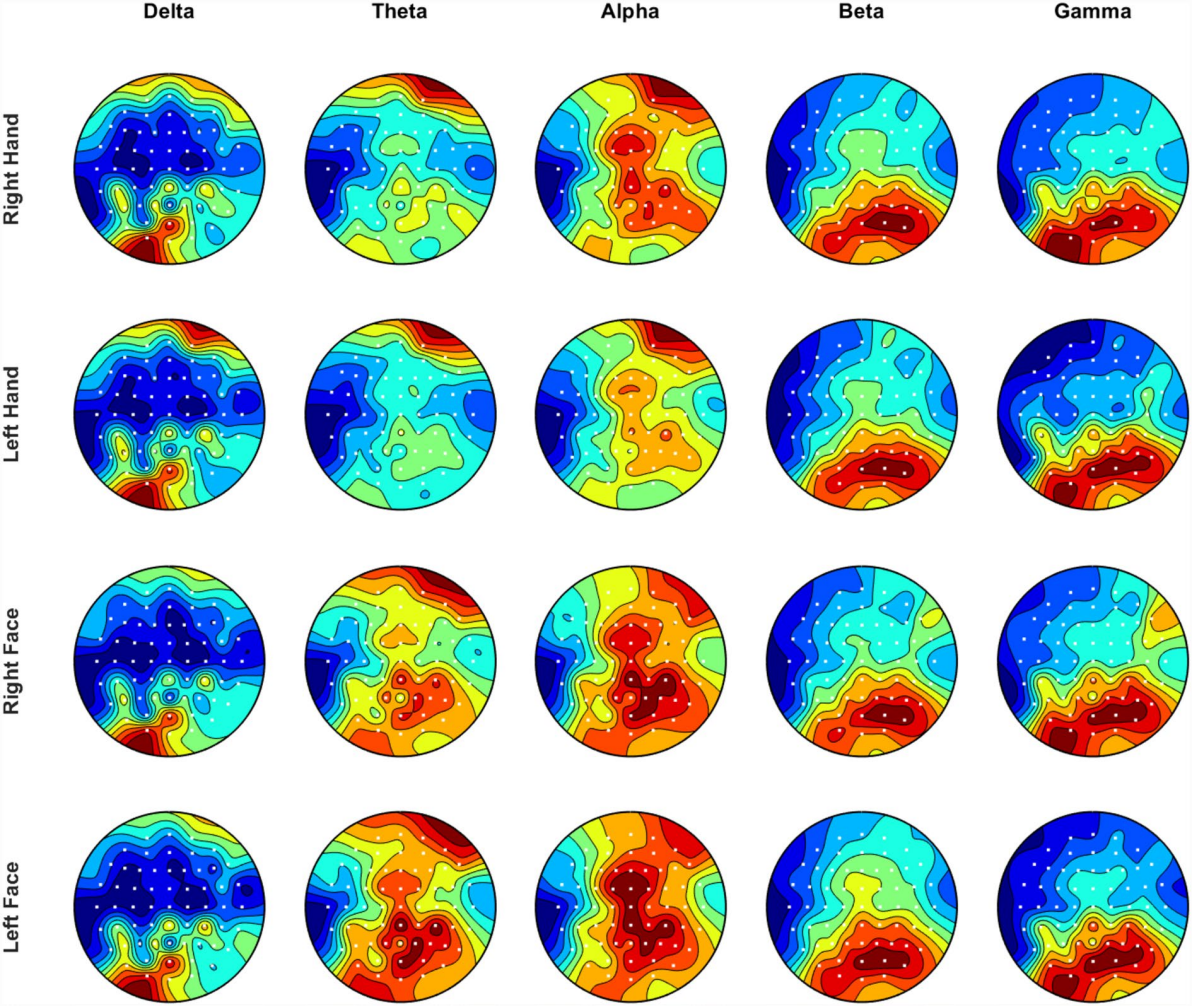
Topography of the control group. The maps were created in MATLAB R2019b (https://www.mathworks.com/) with the subaxis function (link).

Normalized mean WPE of electrodes were calculated to visualize topographic maps of different stimuli. Total wavelet packet energy distribution in sub bands was examined and compared between the groups. Comparison between control and patient groups was carried out with statistical analysis. Also, the correlation of EEG signal pattern was evaluated for the Beta and Gamma bands. Since, the Beta and Gamma bands’ relationship with sensory stimulation has been reported in various studies^41–43^, examinations focused on these bands. Similar recording protocol was used in the fMRI study conducted by Şavklıyıldız et al. (2021). In their study, they observed activity in two areas in the somatosensory cortex for the control group hand stimulus and in one dominant area for the face region. The MNI coordinates of these activities for the hand are [−54 −20 44] and [−42 −34 66], respectively. For the face, the peak intensity was located in the [-56 -18 38] MNI coordinates. C3, C5, CP1, CP3 and CP5 electrodes cover the listed coordinates and analysis were performed. MNI coordinates were defined using the AtlasViewerGUI toolbox^44^. In addition to these electrodes, the electrodes that are symmetrical and the CPZ electrode, which is dominant in the topographic maps (see Fig. 2), were also included in the study. The mean wavelet packet energies of each selected electrode in the control and patient groups were calculated and the EEG signal patterns were evaluated using cross correlation.

### Statistical analysis

Statistical analysis were performed using SPSS (version 23, IBM) program. The Mann Whitney U test was used for pairwise comparison of the patient and control groups.

### Ethical approval

All procedures performed in studies involving human participants were in accordance with the ethical standards of the institutional and/or national research committee (Akdeniz University, Faculty of Medicine, Clinical Research Ethics Committee, 2012-KAEK-20/996, 23.10.2019) and with the 1964 Helsinki declaration and its later amendments or comparable ethical standards.

### Informed consent

Informed consents were obtained from all participants.

## Results

In this study, EEG recordings taken by stimulating the lower part of the face and palm of the control group consisting of 3 full-face transplant patients, 4 patients with full-face lesions and 10 healthy subjects were analyzed using wavelet packet transform. Since the sensorial representation of hand and face on the cortex close to each other, the topographic maps of controls shown in Fig. 2 give similar activation pattern.

**Figure 3:**
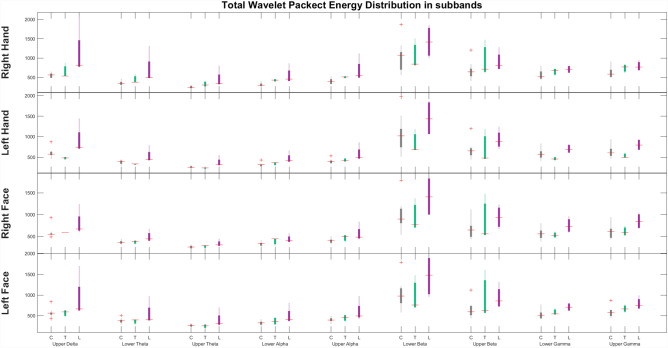
The sub-band total wavelet packet energy (WPE) across different subject groups and stimuli, where the grey, green, and purple boxes denote controls (C), transplants (T), and facial lesions (L) respectively.

As a result of the analysis made on three groups, total wavelet packet energy distribution in sub-bands shown in Fig. 3 with boxplots. There are significant differences between the delta band of the subjects.

**Figure 4:**
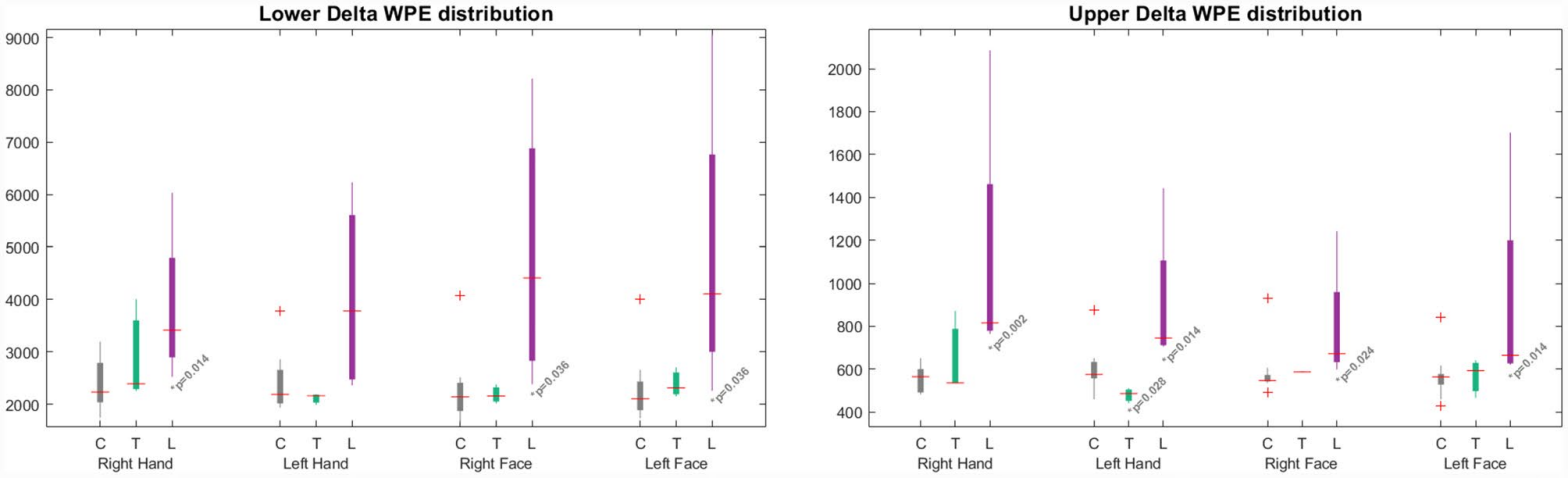
Upper and lower delta band total wavelet packet energy (WPE) distribution for different subject groups and stimuli, where the grey, green, and purple boxes denote controls (C), transplants (T), and facial lesions (L) respectively.

As seen in the Süzen’s previous report, the division of beta bands into two groups with equal frequency as upper and lower bands provide a more precise detection of the activity. It was understood that the beta band activity was dominant in the right cerebral hemisphere according to the topographic maps and had similar results in the differences between the right-hand stimulus and the right lower face stimuli in patients with full face lesions. The reason for this dominance was the upper beta activity in the 21–30 Hz frequency range. Dividing the band into upper and lower sub bands were revealed significant details about the bands^34^. In the ongoing process of the study, especially the differences in the delta band have become an important finding of this study. In contrast to the beta band, energy distribution in the upper delta band clearly reflected the facial lesion patient group. The delta total energy distribution is shown in Fig. 4 with the lower delta. The delta band energy of the transplant patients was close to the control group and the lesion patients are statistically significant (α = 0.05) from the control group. All p-values were given with box plot figures.

Since there was a statistically significant difference in all stimuli between the lesion and control groups in the upper delta, the upper delta band is more dominant in differentiating the patient group than the lower delta band.

**Figure 5:**
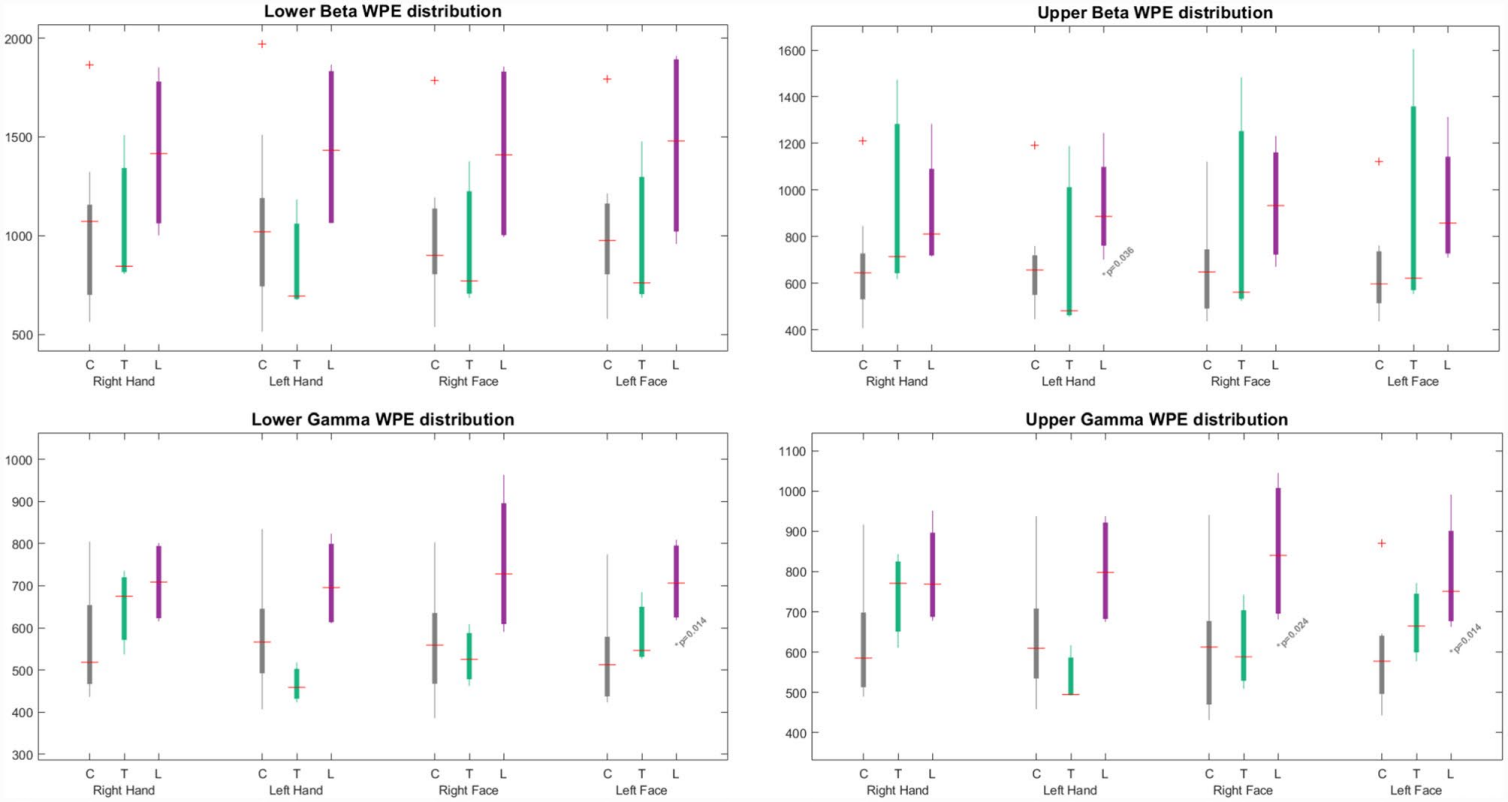
Beta and gamma sub-bands total wavelet packet energy (WPE) distribution for different subject groups and stimuli, where the grey, green, and purple boxes denote controls (C), transplants (T), and facial lesions (L) respectively.

Beta and gamma total energy distribution, with different stimuli and lower–upper band separation are shown in Fig. 5**.** The total energy distribution of the transplant group in beta and gamma bands is not statistically significant compared to the control group. For facial region stimuli, there is a statistically significant difference in the upper gamma distributions of the lesion group compared to the control group.

**Figure 6:**
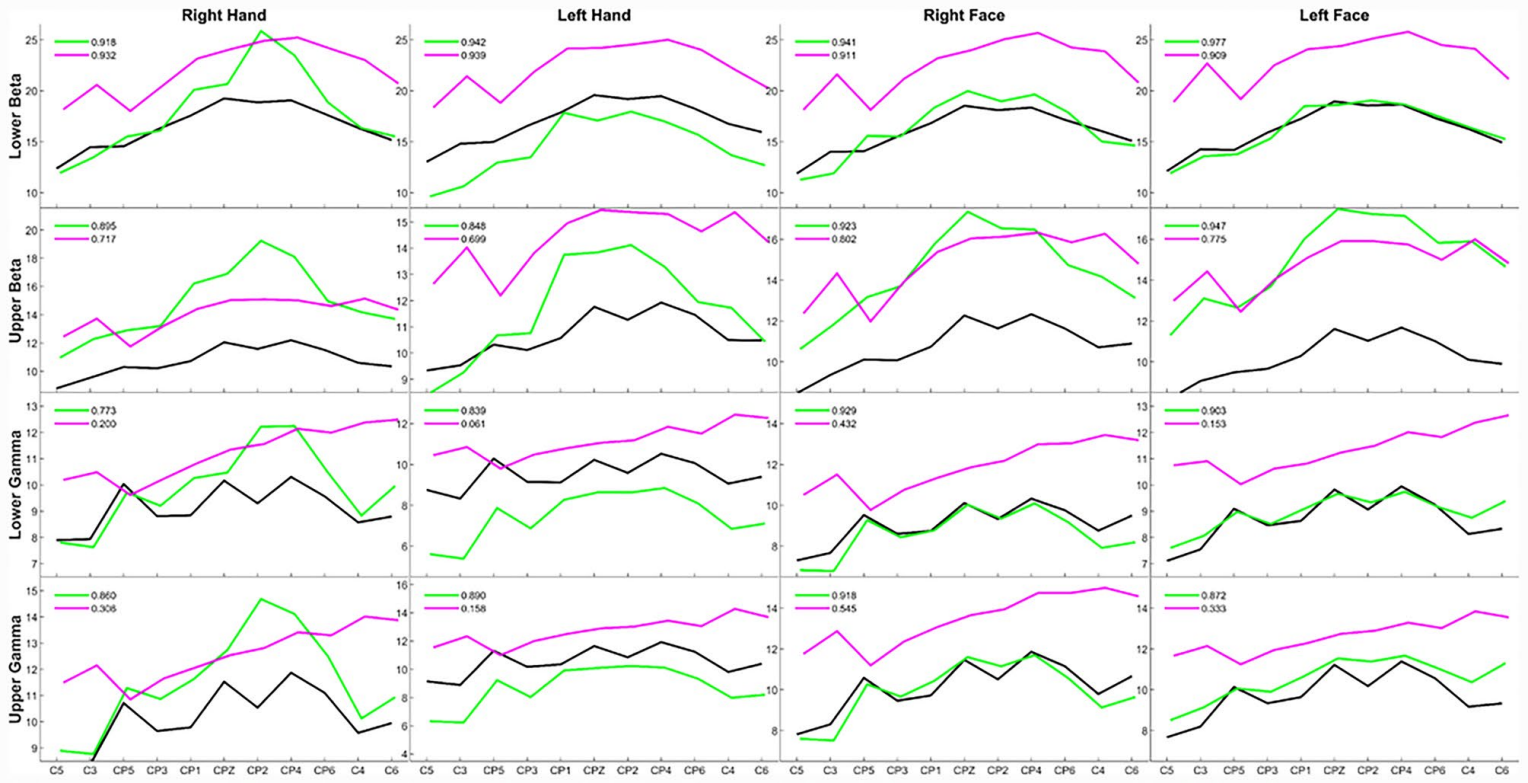
The mean wavelet packet energies of each selected electrode in control (black), transplant (green) and lesion (magenta) groups. The values given in the upper left corner of the relevant figure represent the cross-correlation coefficients between control-transplant (in green) and control-facial lesion (in magenta).

Distribution of energy values in beta and gamma bands was calculated to examine the main effect of these bands. On the other hand, several electrodes were selected with respect to the location of previous studies to inspect the sensory representation of hand and face on beta and gamma bands. The mean wavelet packet energies of each selected electrode are shown in Fig. 6.

The correlation value between the patient and control group for the lower beta band has the highest value. While the amplitudes in the lower beta band of the face region were similar between the transplant and the control groups, the lesion group had higher amplitudes. In the upper beta face region, the amplitudes of the patient groups were relatively higher than the control group. The difference between the patient groups in the upper beta and lower beta face region also highlights the importance of the separation in dividing the beta band into sub bands.

While the lower and upper gamma band electrode activity patterns of the face region have activity at close amplitudes and high correlation for the transplant group and the healthy group, the lesion group patterns have higher amplitudes and low correlation. Like the face region, the hand region has similar lower and upper gamma band electrode activity patterns. However, the activity correlations between both patient groups and the control group were lower than the face region correlation values.

## Discussion

In this study, we focused on the analysis of EEG spectrum and the detection of the plastic changes in EEG of the full-face transplantation. Electroencephalography (EEG) measures the brain’s electric fields non-invasively, focusing on postsynaptic potentials from pyramidal neurons. EEG oscillations at the scalp capture a subset of brain activity. Low-frequency oscillations (e.g., delta) span larger neural populations, while high-frequency oscillations (e.g., beta) involve smaller neural assemblies^45^. Delta waves are characterized by high amplitude and are the slowest brainwaves (0.5–4 Hz). Delta waves are crucial for restorative sleep, facilitating healing and growth processes^46^. Beta oscillations (13–30 Hz) relate to mental states such as concentration and anxiety, representing an excitatory mechanism. Gamma oscillations (30–90 Hz) are associated with arousal. Gamma activity involves a sequence of excitation and inhibition. After a strong stimulus the gamma cycle starts. It has been shown that the gamma cycle contains a characteristic sequence of excitation and inhibition^47,48^.

The main challenge in this study is the small number of people suitable for patient groups. Examination of the brain activity of such patients is not widely performed in the clinic. For this reason, details of the pre-transplant brain activities of such patients are limited considering the number of full-face transplants in the world over roughly two decades. Facial lesion patients have lived with wounds for a long period of time. Full-face transplant patients had also lived a similar period of their lives with facial lesions. Although, in our study, the number of patients was small and pre-transplant EEG recordings were not available, the results of the lesion patients can provide information about the previous status of the transplant patients.

Siemionow et al. conducted a study with a single face-transplant patient. When the EEG-focused cortical responses resulting from sensory tactile stimulation of the patient with facial lesion who had a gunshot wound were examined before and after transplantation, the beta band strength value was higher (38.8 nAm^2^) in the first 3 months after surgery compared to the pre-transmission value (18.2 nAm^2^/Hz). After a few months the power value decreased, but still (8–15 nAm^2^/Hz) above the control group^26^. According to the study, it has been reported that beta band power changes reflect the post-transplant recovery process.

Differences in lower and upper gamma band electrode activity patterns and patient-control correlation values may indicate complementary phenomena between the hand and face^14^. It is considered that the representation areas of the lost or denervated sensory tissues in the brain are used by the surrounding regions over time and that they expand (or invade) to these regions. Reduction of this expansion is an important parameter in the recovery of transplant patients. It is necessary for the functionality of the tissue to reach its optimal state.

Our findings indicate that the upper delta amplitude values of hand and face stimuli in EEG are effective in revealing the differences in the cortical restructuring process of face transplant and facial lesion patients. Delta waves are the most prominent EEG feature of human non-rapid eye movement sleep, which has its origin in cortical layers. Delta waves are proposed as sensors of sleep-dependent synaptic plasticity^49^. The strong relationship between increased delta activity during sleep and synaptic plasticity has been carried to another dimension in different studies conducted recently. Consistent with Tononi's Synaptic Homeostasis Hypothesis, delta waves can also increase while the person is awake, apart from 'sleep-related synaptic plasticity'. The clearest answer to the question of how the increased delta activity in conscious individuals can be explained from studies emphasizing that the 'shift' to delta waves in EEG activity actually means 'neuronal restructuring', not a dysfunction^46,50^. In these studies, the presence of delta waves recorded in wakefulness has been shown to be an ongoing reorganization of the cortex in both acute and chronic processes. Chronic pain after facial lesion causes central plastic changes^51^. The delta wave asymmetry and increase in intensity seen in patients with facial lesions and face transplants can also be explained by the intense central plasticity. Facial injuries, both burns and post-traumatic cause a unique pain syndrome because of its multiple components and its altering pattern over time. Facial pain is exceptional because care involves repeated traumas or manipulations of the injured sites.

As a result of this study, the differences in delta waves show a sign of cortical plasticity for traumas involving different limb losses. These findings raise the question of whether the delta band can be used as a marker in the evaluation of the post-transplant healing process. We believe that this issue should be explored in future studies.

## Data Availability

Sequence data that support the findings of this study have been deposited in the Mendeley Data which is an open data repository (link)^52^ and OneDrive (link) All other additional data are available from the corresponding author upon request.
